# Supplementary material for: Fidelity and adherence to a liquefied petroleum gas stove and fuel intervention: The multi-country Household Air Pollution Intervention Network (HAPIN) trial
Source: Environ Int. 2023 Sep;179:108160. doi: 10.1016/j.envint.2023.108160 (PMC10512198; doi:10.1016/j.envint.2023.108160)
Supplement: Supplementary data 1 [file mmc1.pdf]

**Supplemental Materials**

Figure S1. Number of participants receiving a stove repair out of all enrolled participants by month. Dashed line indicates onset of the global COVID-19 pandemic (March 17, 2020).

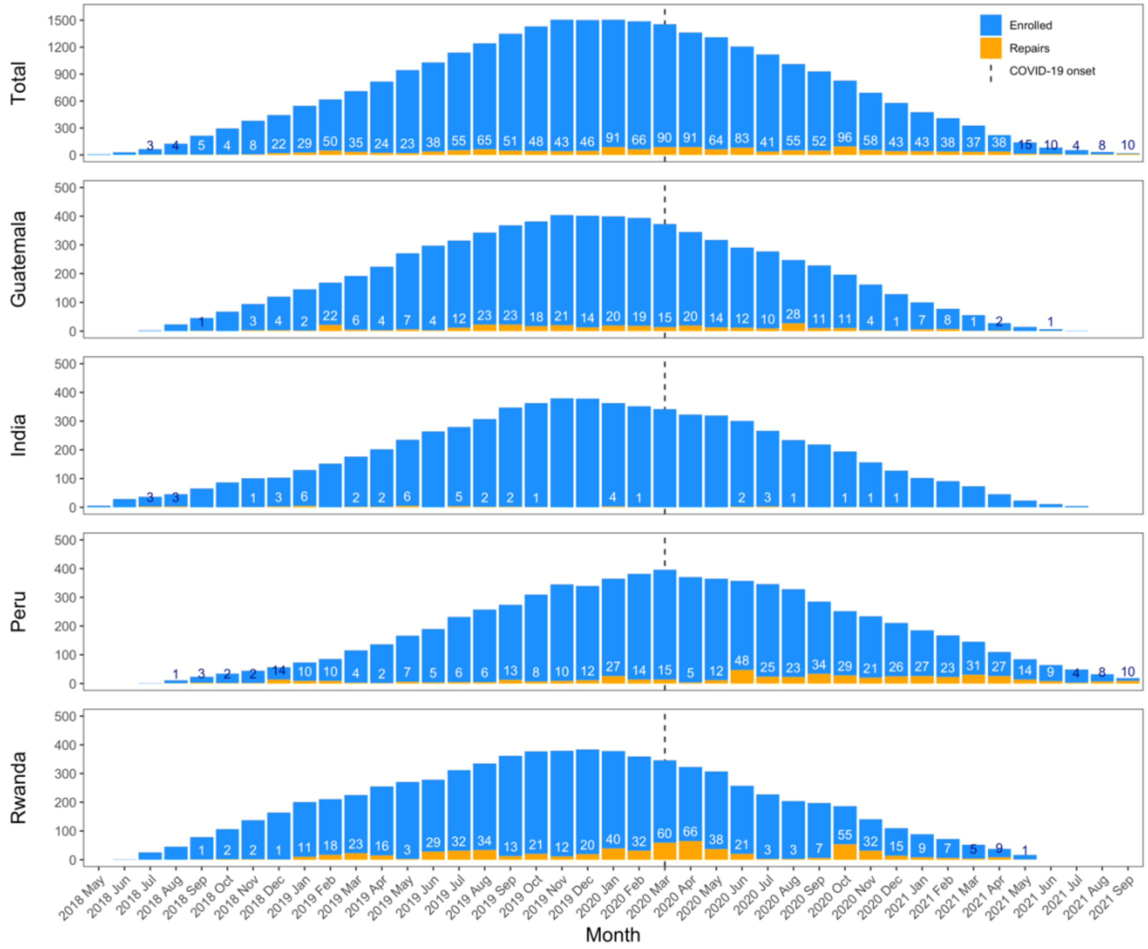

Figure S2. Frequency of the percent of stove-use-monitored days in which traditional stove use (TSU) was detected via stove use monitors (SUMs) in intervention households during the pregnancy period.

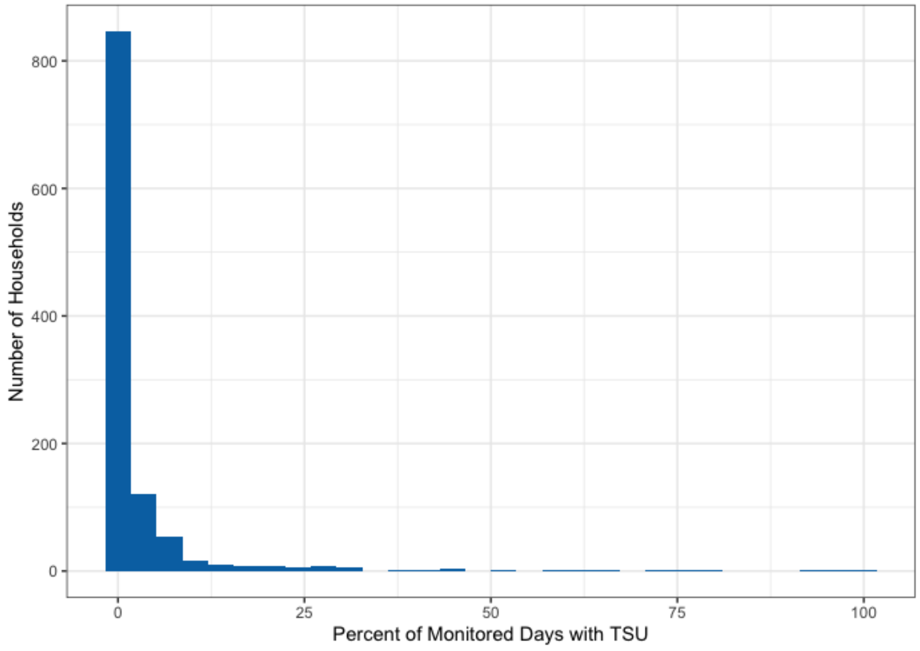

Figure S3. Frequency of the percent of stove-use-monitored days in which traditional stove use (TSU) was detected via stove use monitors (SUMs) in intervention households during the post-birth or infancy period.

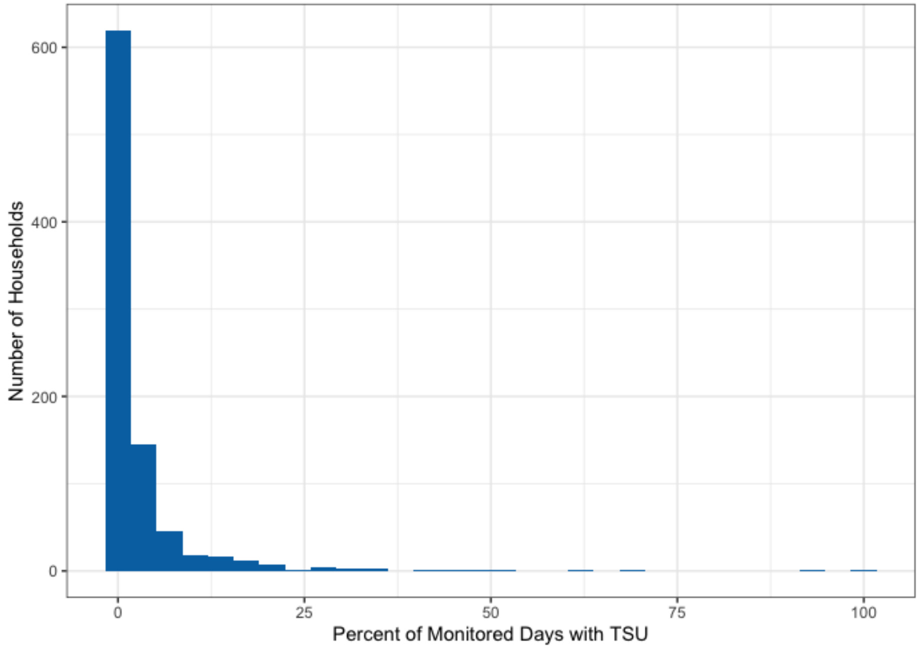

Figure S4. Percent of intervention households who destroyed their traditional stove at LPG stove installation and have no subsequent SUMs data (blue), had zero traditional stove use (TSU) according to SUMs data (sepia), or had less than one day of TSU per month (red), by country and overall. Percentages displayed in white represent the total percent of participants falling into either of these three categories. Grey bars represent participants with one or more day with traditional stove use per month (dark grey) or who retained their traditional stove but are missing SUMs data (light grey).

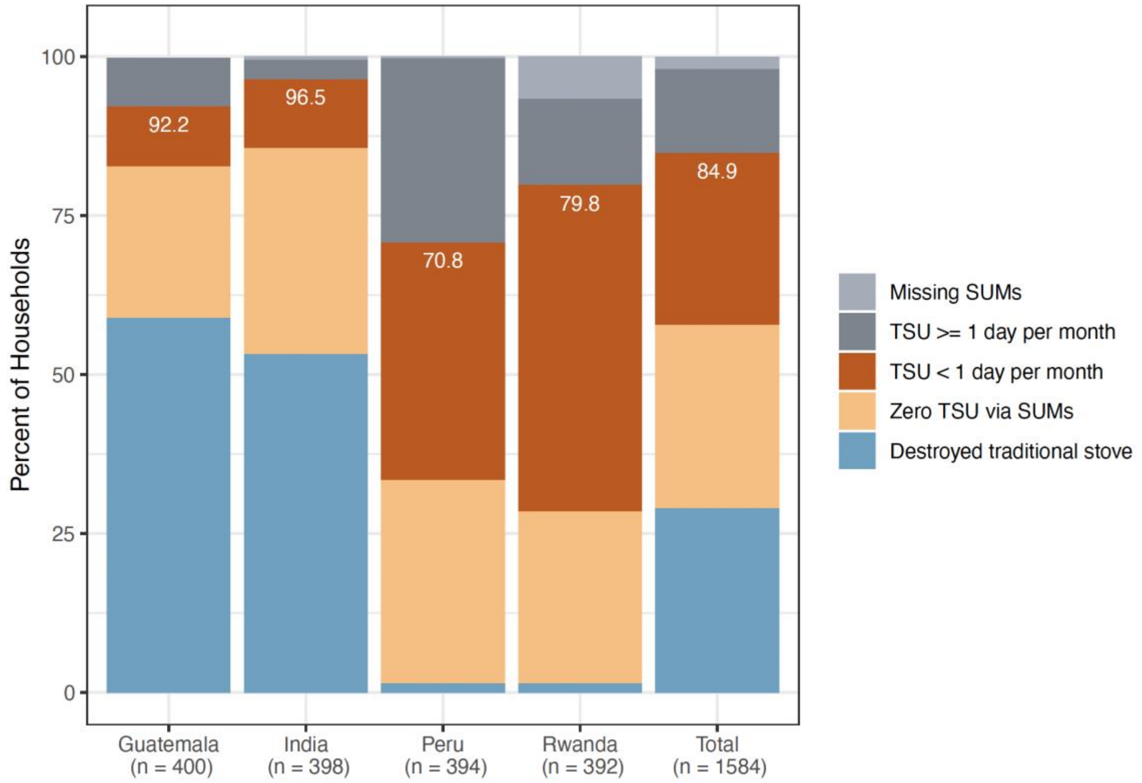

Table S1. Reports of general problems or concerns with the LPG stove and running out of LPG at behavioral reinforcement visits in pregnancy (“preg”), the post-birth or infancy period (“infcy”), and total across the full trial (“full”) among intervention participants.

| | Guatemala | | | India | | | Peru | | | Rwanda | | | Total | | |
|---|---|---|---|---|---|---|---|---|---|---|---|---|---|---|---|
| Study period in relation to the baby’s birth | Preg | Infcy | Full | Preg | Infcy | Full | Preg | Infcy | Full | Preg | Infcy | Full | Preg | Infcy | Full |
| N Intervention Participants | 400 | 382 | 400 | 398 | 384 | 398 | 394 | 379 | 394 | 392 | 372 | 392 | 1584 | 1517 | 1584 |
| # (%) of intervention participants who indicated a problem or concern about LPG use at any reinforcement visit | 58 (15) | 145 (38) | 169 (42) | 3 (1) | 0 | 3 (1) | 146 (37) | 140 (37) | 211 (54) | 68 (17) | 291 (78) | 302 (77) | 275 (17) | 576 (38) | 685 (43) |
| Median (Q1, Q3) # of reinforcement visits in which participant indicated a problem or concern per participant, among those with >=1 problems/ concerns | 1 (1, 1) | 1 (1, 2) | 1 (1, 2) | 1 (1, 1) | 0 | 1 (1, 1) | 1 (1, 2) | 2 (1, 3) | 2 (1, 3) | 1 (1, 1) | 2 (1, 3) | 2 (1, 3) | 1 (1, 2) | 2 (1, 3) | 2 (1, 3) |

| | | | | | | | | | | | | | | | |
|---|---|---|---|---|---|---|---|---|---|---|---|---|---|---|---|
| # (%) of intervention participants who reported running out of LPG at any reinforcement visit | 4 (1) | 39 (10) | 42 (11) | 0 | 0 | 0 | 27 (7) | 91 (24) | 105 (27) | 40 (10) | 255 (69) | 263 (67) | 71 (4) | 385 (25) | 410 (26) |
| Median # (Q1, Q3) of reinforcement visits in which participant reported running out of LPG per participant, among those with >=1 instance of running out | 1.0 (1, 1.5) | 1.0 (1, 1) | 1.0 (1, 1) | 0 | 0 | 0 | 1.0 (1, 1) | 1.0 (1, 2) | 1.0 (1, 2) | 1.0 (1,1) | 2.0 (1, 2) | 2.0 (1, 2) | 1.0 (1, 1) | 1.0 (1, 2) | 1.0 (1, 2) |

Reported reasons for occasional use of the traditional stove were similar in pre-birth and post-birth periods, except for running out of LPG, which was more common in the post-birth compared to the pre-birth period in Guatemala, Peru, and Rwanda (likely driven by the higher frequency of running out of LPG in the early months of the COVID-19 pandemic, see Figure 1). In Rwanda, reported challenges with cleaning or maintaining the LPG stove or the stove not functioning correctly also increased post-birth compared to pre-birth.

Table S2. Types of repairs made to LPG equipment by country and overall.

| | Guatemala | India | Peru | Rwanda | Total |
|---|---|---|---|---|---|
| Total repair visits made | 348 | 50 | 547 | 641 | 1586 |
| Types of repairs | | | | | |
| Stove* | 208 (60%) | 32 (64%) | 443 (81%) | 611 (95%) | 1294 (82%) |
| Knobs/burners | 23 (7%) | 23 (46%) | 309 (56%) | 513 (80%) | 868 (55%) |
| Stove valves | 187 (54%) | 7 (14%) | 113 (21%) | 4 (1%) | 311 (20%) |
| Stove or part replaced | 1 (0.3%) | 3 (6%) | 41 (8%) | 90 (14%) | 135 (9%) |
| LPG cylinder/regulator | 3 (1%) | 13 (26%) | 54 (10%) | 13 (2%) | 83 (5%) |
| Hose and connectors | 1 (0.3%) | 7 (14%) | 127 (23%) | 8 (1%) | 143 (9%) |
| Switch valve | 139 (40%) | N/A | N/A | 12 (2%) | 151 (15%) |

*Only includes most commonly reported stove problems; a few “other” responses not summarized here
NOTE: Numbers may add up to greater than 100% because multiple problems may have been fixed at the same visit.

Repairs to the stove were most common, with repairs to the knobs or burners being most frequent in India, Peru, and Rwanda and repairs to the stove valves most frequent in Guatemala. Repairs to the switch valve (which was only used in Guatemala and Rwanda, to make it easier for households to switch from one cylinder of LPG when it became empty to the other one in the household) were much more common in Guatemala than in Rwanda. Repairs to the LPG cylinder or regulator were most common in India, and repairs to the hose connecting the cylinder to the stove were most common in Peru. Most repairs were completed on the same day as identification or request; the median (Q1, Q3) number of days between identification or request for a repair and successful completion of the repair was 0 (0, 0). Among the 1,563 intervention participants in which observation of the LPG stove was done at the conclusion of their participation in the trial, most LPG stoves were observed to be functioning properly (98.5%). Of the 23 stoves that were not functioning properly, the main two burners were still functioning in 19 (83%), and the 4 with non-working burners were repaired prior to the participant’s exit from the study (data not shown).

Table S3. Observations of traditional stove use and follow-up behavioral reinforcement in pregnancy ("preg"), the post-birth or infancy period ("infcy"), and total across the full trial ("full") among intervention participants.

| | Guatemala | | | India | | | Peru | | | Rwanda | | | Total | | |
|---|---|---|---|---|---|---|---|---|---|---|---|---|---|---|---|
| Study period in relation to the baby's birth | Preg | Infcy | Full | Preg | Infcy | Full | Preg | Infcy | Full | Preg | Infcy | Full | Preg | Infcy | Full |
| Total observations made | 7012 | 19276 | 26288 | 2766 | 7079 | 9845 | 3360 | 8086 | 11446 | 2571 | 5091 | 7662 | 15709 | 39532 | 55241 |
| n (%) of observation visits with traditional stove use observed | 135 (2) | 413 (2) | 548 (2) | 3 (0.1) | 2 (0.03) | 5 (0.1) | 173 (5) | 347 (4) | 520 (5) | 131 (5) | 302 (6) | 433 (6) | 442 (3) | 1064 (3) | 1506 (3) |
| n (%) of traditional stoves that were observed to be recently used with no SUMs installed | 97 (72) | 309 (75) | 406 (74) | 0 | 1 (50) | 1 (20) | 17 (10) | 41 (12) | 58 (11) | 13 (10) | 57 (19) | 70 (16) | 127 (29) | 408 (38) | 535 (36) |
| n (%) of traditional stove use observations with a reinforcement | - | - | 420 (77) | - | - | 2 (40) | - | - | 514 (99) | - | - | 410 (95) | - | - | 1346 (89) |

| | | | | | | | | | | | | | | | |
|---|---|---|---|---|---|---|---|---|---|---|---|---|---|---|---|
| visit at a later date | | | | | | | | | | | | | | | |
| Median (Q1, Q3) days between traditional stove use observation and reinforcement visit | - | - | 26.5 (10, 179.5) | - | - | 33.5 (3, 64) | - | - | 0 (0, 0) | - | - | 19 (0, 72) | - | - | 9 (0, 56) |

Table S4. Stove use monitoring in intervention households by country.

| | Guatemala | India | Peru | Rwanda | Total |
|---|---|---|---|---|---|
| **# Intervention households who received LPG stove** | **400** | **398** | **394** | **392** | **1584** |
| # Households who voluntarily removed TS at LPG delivery and did not re-install it during pregnancy (%) | 265 (66) | 214 (54) | 8 (2) | 10 (3) | 497 (31) |
| # Households missing SUMs data during pregnancy for unknown reason* (%) | 2 (0.5) | 2 (0.5) | 1 (0.3) | 43 (11) | 48 (3) |
| **Total households with valid SUMs during pregnancy** | **133** | **182** | **385** | **339** | **1039** |
| # Households with miscarriage or stillbirth | 16 | 10 | 9 | 13 | 48 |
| # Households with infant death or drop-out <14 days after birth | 2 | 4 | 6 | 7 | 19 |
| **# live births with post-birth follow-up of mother >14 days** | **382** | **384** | **379** | **372** | **1517** |
| # Households who removed TS at LPG delivery and did not re-install it during pregnancy or post-birth (%) | 222 (58) | 204 (53) | 5 (1) | 5 (1) | 436 (29) |
| # Households who removed TS during pregnancy or <14 days after birth and did not re-install post-birth (%) | 0 | 28 (7) | 33 (9) | 0 | 61 (4) |
| # Households missing SUMs data post-birth for unknown reason* (%) | 58 (15) | 13 (3) | 37 (10) | 35 (9) | 143 (9) |
| **Total households with valid SUMs post-birth** | **102** | **139** | **304** | **332** | **877** |
| | | | | | |
| **Total households with valid SUMs in pregnancy and/or post-birth periods** | **164** | **184** | **387** | **360** | **1095** |

TS=Traditional stove; SUMs=Stove use monitors; LPG=Liquefied petroleum gas

*Reasons for missing SUMs data may include SUM device errors, participant manipulation or removal of the SUMs, participant misclassification (i.e., failure to record removal of a traditional stove, or mistakenly indicating that participant retained a traditional stove when they had removed it), fieldworker errors in SUM installation, inability to match SUMs data to a household, among others.

Table S5. Additional details on traditional stove monitoring and traditional stove use (TSU) based on SUMs data from intervention households in pregnancy ("preg"), the post-birth or infancy period ("infcy"), and total across the full trial ("full"). This table complements the data in Table 6 of the main paper.

| | Guatemala | | | India | | | Peru | | | Rwanda | | | Total | | |
|---|---|---|---|---|---|---|---|---|---|---|---|---|---|---|---|
| Study period in relation to the baby's birth | Preg | Infcy | Full | Preg | Infcy | Full | Preg | Infcy | Full | Preg | Infcy | Full | Preg | Infcy | Full |
| Total households with valid SUMs data | 133 | 102 | 164 | 182 | 139 | 184 | 385 | 304 | 387 | 339 | 332 | 360 | 1039 | 877 | 1095 |
| Days with stove-use-monitoring per household: median (Q1, Q3) | 99 (29, 146) | 358 (153, 393) | 169.5 (41, 501) | 127 (91, 149.5) | 366 (315.5, 376) | 460 (156.5, 510) | 145 (121, 170) | 370 (205, 384) | 471 (196.5, 538) | 134 (95, 161) | 338 (244, 387) | 451.5 (328, 511) | 134 (97.5, 161) | 363 (234, 385) | 435 (193, 521) |
| Percent of monitored days with TSU detected: mean (range) | 3.8 (0 - 95.2) | 6.8 (0 - 100) | 4.6 (0 - 94) | 1.5 (0 - 53.2) | 0.3 (0 - 6.1) | 1.1 (0 - 53.2) | 4.3 (0 - 81) | 3.6 (0 - 51.8) | 4.3 (0 - 81) | 1.8 (0 - 94.1) | 2.1 (0 - 41.6) | 1.9 (0 - 31.5) | 2.9 (0 - 95.2) | 2.9 (0 - 100) | 3.0 (0 - 94) |
| Households with no SUM-detected TSU: N (%) | 105 (78.9) | 44 (43.1) | 96 (58.5) | 143 (78.6) | 113 (81.3) | 129 (70.1) | 177 (46) | 145 (47.7) | 126 (32.6) | 194 (57.2) | 114 (34.3) | 106 (29.4) | 619 (59.6) | 416 (47.4) | 457 (41.7) |

| | | | | | | | | | | | | | | | |
|---|---|---|---|---|---|---|---|---|---|---|---|---|---|---|---|
| Households with < 1 day with TSU per 30 days of monitoring: N (%) | 117 (88.0) | 72 (70.6) | 133 (81.1) | 167 (91.8) | 135 (97.1) | 172 (93.5) | 288 (74.8) | 228 (75) | 273 (70.5) | 293 (86.4) | 274 (82.5) | 307 (85.3) | 865 (83.3) | 709 (80.8) | 885 (80.8) |

Table S6. Traditional stove use (TSU) based on SUMs data from the subset of intervention households with an enrolled non-pregnant adult woman (40-79 years old) across the full trial.

| | Guatemala | India | Peru | Rwanda | Total |
|---|---|---|---|---|---|
| **# Intervention households with an enrolled non-pregnant adult woman and SUMs monitoring** | **39** | **28** | **62** | **14** | **143** |
| Days with stove-use-monitoring per household: median (Q1, Q3) | 223.0 (73.0, 526.5) | 459.0 (296.2, 531.5) | 509.0 (306.8, 538.5) | 335.0 (280.0, 464.5) | 465.0 (206.0, 533.0) |
| Proportion of follow-up time monitored by SUMs: median (Q1, Q3) | 49.1 (18.8. 96.4) | 98.4 (62.4, 99.8) | 97.7 (65.2, 99.8) | 59.0 (50.8, 86.5) | 92.7 (43.9, 99.8) |
| Percent of monitored days with TSU detected: median (Q1, Q3) | 0.3 (0.0, 10.3) | 0.0 (0.0, 0.5) | 1.3 (0.2, 4.9) | 1.0 (0.0, 2.7) | 0.4 (0.0, 3.6) |
| Households with no SUM-detected TSU: N (%) | 17 (43.6) | 18 (64.3) | 13 (21.0) | 5 (35.7) | 53 (37.1) |
| Avg # days with TSU per 30 days of monitoring: median (Q1, Q3) | 0.1 (0.0, 3.1) | 0.0 (0.0, 0.1) | 0.4 (0.1, 1.5) | 0.3 (0.0, 0.8) | 0.1 (0.0, 1.1) |
| Households with < 1 day with TSU per 30 days of monitoring: N (%) | 24 (61.5) | 27 (96 4) | 42 (67.7) | 12 (85.7) | 105 (73.4) |

Table S7. Traditional stove use based on SUMs data from intervention households pre-COVID-19 and post-COVID-19.

| | Guatemala | | India | | Peru | | Rwanda | | Total | |
|---|---|---|---|---|---|---|---|---|---|---|
| | Pre-COVID | Post-COVID | Pre-COVID | Post-COVID | Pre-COVID | Post-COVID | Pre-COVID | Post-COVID | Pre-COVID | Post-COVID |
| Total households with valid SUMs data | 163 | 74 | 184 | 99 | 383 | 258 | 357 | 226 | 1087 | 657 |
| Days with stove-use-monitoring per household: median (Q1, Q3) | 89 (38, 302) | 132 (72.2, 220.5) | 247.5 (121, 349.2) | 196.0 (114.5, 263) | 181 (90, 300.5) | 218.5 (133.2, 354.8) | 292 (192, 399) | 162 (89.2, 239.5) | 225 (110.5, 350) | 188 (108, 279) |
| Proportion of follow-up time monitored by SUMs: median (Q1, Q3) | 30.5 (11.9, 95.9) | 98.5 (94.2, 99.3) | 96.8 (44.7, 100) | 99.2 (97.5, 99.6) | 100 (72.5, 100) | 99.2 (65.9, 99.6) | 91.4 (71.7 100) | 89.3 (65.3, 99.2) | 95.9 (52.4, 100) | 98.7 (72.9, 99.5) |
| Percent of monitored days with TSU detected: median (Q1, Q3) | 0.0 (0.0, 0.9) | 0.0 (0.0, 4.2) | 0.0 (0.0, 0.0) | 0.0 (0.0, 0.0) | 0.2 (0.0, 2.3) | 0.7 (0.0, 5.5) | 0.4 (0.0, 1.6) | 0.7 (0.0, 3.5) | 0.0 (0.0, 1.6) | 0.0 (0.0, 3.4) |
| Households with no SUM-detected TSU: N (%) | 106 (65) | 39 (52.7) | 140 (76.1) | 82 (82.8) | 190 (49.6) | 110 (42.6) | 144 (40.3) | 98 (43.4) | 580 (53.4) | 329 (50.1) |
| Avg # days with TSU per 30 days of monitoring: median (Q1, Q3) | 0.0 (0.0, 0.3) | 0.0 (0.0, 1.3) | 0.0 (0.0, 0.0) | 0.0 (0.0, 0.0) | 0.1 (0.0, 0.7) | 0.2 (0.0, 1.6) | 0.1 (0.0, 0.5) | 0.2 (0.0, 1.1) | 0.0 (0.0, 0.5) | 0.0 (0.0, 1.0) |
| Households with < 1 day with TSU per 30 days of monitoring: N (%) | 135 (82.8) | 52 (70.3) | 171 (92.9) | 98 (99) | 303 (79.1) | 175 (67.8) | 315 (88.2) | 164 (72.6) | 924 (85) | 489 (74.4) |

Table S8. Traditional stove use based on SUMs data from intervention households in early-COVID-19 (March 17, 2020 – July 17, 2020) and late-COVID-19 (after July 17, 2020) periods.

| | Guatemala | | India | | Peru | | Rwanda | | Total | |
|---|---|---|---|---|---|---|---|---|---|---|
| | Early-COVID | Late-COVID | Early-COVID | Late-COVID | Early-COVID | Late-COVID | Early-COVID | Late-COVID | Early-COVID | Late-COVID |
| Total households with valid SUMs data | 74 | 35 | 99 | 63 | 258 | 192 | 225 | 146 | 656 | 436 |
| Days with stove-use-monitoring per household: median (Q1, Q3) | 120.5 (72.2, 122) | 118 (71, 196) | 122 (114.5, 122) | 122 (81, 206.5) | 122 (118.2, 122) | 146 (69, 279.8) | 120 (77, 122) | 104 (62, 188) | 122 (99, 122) | 124 (69.8, 224) |
| Proportion of follow-up time monitored by SUMs: median (Q1, Q3) | 98.8 (59.2, 100) | 98.4 (92.1, 99) | 100 (93.9, 100) | 98.9 (98.5, 99.4) | 100 (96.9, 100) | 98.8 (93.5, 99.5) | 98.4 (63.1, 100) | 83.9 (51.2, 99) | 100 (81.1, 100) | 98.5 (77, 99.3) |
| Percent of monitored days with TSU detected: median (Q1, Q3) | 0.0 (0.0, 3.4) | 0.0 (0.0, 1.9) | 0.0 (0.0, 0.0) | 0.0 (0.0, 0.0) | 0.0 (0.0, 7.4) | 0.0 (0.0, 2.7) | 0.0 (0.0, 4.2) | 0.0 (0.0, 1.5) | 0.0 (0.0, 3.7) | 0.0 (0.0, 1.5) |
| Households with no SUM-detected TSU: N (%) | 48 (64.9) | 22 (62.9) | 86 (86.9) | 57 (90.5) | 136 (52.7) | 112 (58.3) | 113 (50.2) | 95 (65.1) | 383 (58.4) | 286 (65.6) |
| Avg # days with TSU per 30 days of monitoring: median (Q1, Q3) | 0.0 (0.0, 1) | 0.0 (0.0, 0.6) | 0.0 (0.0, 0.0) | 0.0 (0.0, 0.0) | 0.0 (0.0, 2.2) | 0.0 (0.0, 0.8) | 0.0 (0.0, 1.3) | 0.0 (0.0, 0.5) | 0.0 (0.0, 1.1) | 0.0 (0.0, 0.4) |
| Households with < 1 day with TSU per 30 days of monitoring: N (%) | 55 (74.3) | 29 (82.9) | 96 (97) | 62 (98.4) | 175 (67.8) | 151 (78.6) | 163 (72.4) | 125 (85.6) | 489 (74.5) | 367 (84.2) |

Table S9. Percent of intervention participants with observations of traditional stove use during pregnancy (“preg”), post-birth or infancy (“infcy”), and total across the full trial (“full”), and extent to which traditional stove use was observed per participant.

| | Guatemala | | | India | | | Peru | | | Rwanda | | | Total | | |
|---|---|---|---|---|---|---|---|---|---|---|---|---|---|---|---|
| Study period in relation to the baby’s birth | Preg | Infcy | Full | Preg | Infcy | Full | Preg | Infcy | Full | Preg | Infcy | Full | Preg | Infcy | Full |
| N intervention participants | 400 | 382 | 400 | 398 | 384 | 398 | 394 | 379 | 394 | 392 | 372 | 392 | 1584 | 1517 | 1584 |
| n (%) of intervention participants who received an observation visit | 399 (99.8) | 382 (100) | 399 (99.8) | 396 (99.5) | 384 (100) | 396 (99.5) | 393 (99.8) | 379 (100) | 393 (99.8) | 375 (95.7) | 369 (99.2) | 387 (98.7) | 1563 (98.7) | 1514 (99.8) | 1575 (99.4) |
| Median (Q1, Q3) # of observation visits, out of all intervention participants | 17 (13, 21) | 49.5 (39, 60) | 65 (52, 80) | 7 (6, 8) | 19 (17, 21) | 26 (22, 29) | 9 (7, 10) | 22 (20, 24) | 30 (28, 32) | 7 (4, 9) | 14 (9, 19) | 22 (14, 26) | 9 (6, 11) | 21 (17, 26) | 29 (23, 34) |
| n (%) of intervention participants with any observation of traditional stove use | 71 (18) | 204 (53) | 224 (56) | 3 (0.8) | 2 (0.5) | 5 (1.3) | 112 (29) | 150 (40) | 202 (51) | 85 (23) | 193 (52) | 229 (58) | 271 (17) | 549 (36) | 660 (42) |

| | | | | | | | | | | | | | | | |
|---|---|---|---|---|---|---|---|---|---|---|---|---|---|---|---|
| (out of those with any observation visit) | | | | | | | | | | | | | | | |
| Median (Q1, Q3) # of traditional stove use observations per participant, out of those with any TSU observations | 1.0 (1, 2) | 1.0 (1, 2) | 1.0 (1, 2) | 1.0 (1, 1) | 1.0 (1, 1) | 1.0 (1, 1) | 1.0 (1, 2) | 2.0 (1, 3) | 2.0 (1, 3) | 1.0 (1, 2) | 1.0 (1, 2) | 1.0 (1, 2) | 1.0 (1, 2) | 1.0 (1, 2) | 2.0 (1, 3) |
| Median (Q1, Q3) % of observations with TSU observed (among those with any TSU observations) | 6.5 (4.5, 11.1) | 2.5 (1.9, 4.1) | 2.1 (1.5, 3.7) | 14.3 (14.3, 16.7) | 8.4 (4.3, 12.5) | 3.7 (3.4, 3.7) | 14.3 (11.1, 26.1) | 8.3 (4.8, 14.3) | 6.5 (3.3, 11.5) | 16.7 (12.5, 28.6) | 9.1 (5.9, 14.3) | 7.1 (4.3, 11.8) | 12.5 (9.1, 25.0) | 5.4 (3.3, 11.1) | 4.5 (3.0, 9.1) |

Table S10. Observations of traditional stove use in intervention households missing SUMs data.

| | Guatemala | India | Peru | Rwanda | Total |
|---|---|---|---|---|---|
| **# Intervention households who received LPG stove** | **400** | **398** | **394** | **392** | **1584** |
| n (%) intervention households with no SUMs monitoring during pregnancy | 269 (67) | 216 (54) | 9 (2) | 53 (14) | 547 (35) |
| Of those with no SUM in pregnancy, n (%) who received any observation visit during pregnancy | 268 (99.6) | 216 (100) | 8 (89) | 39 (74) | 531 (97) |
| Median (Q1, Q3) number of observation visits conducted during pregnancy among those with no SUMs and >=1 observation visit completed | 16 (13, 20) | 7 (5, 8) | 5.5 (2.5, 9) | 2 (1, 5) | 10 (6, 16) |
| Median (Q1, Q3) # of observations of TSU among those with no SUMs and >=1 observation visit completed | 0 (0, 0) | 0 (0, 0) | 0 (0, 0) | 0 (0, 0) | 0 (0, 0) |
| | | | | | |
| **# Intervention households followed post-birth** | **382** | **384** | **379** | **372** | **1517** |
| n (%) intervention households with no SUMs monitoring post-birth | 280 (73) | 245 (64) | 75 (20) | 40 (11) | 640 (42) |
| Of those with no SUM post-birth, n (%) who received any observation visit post-birth | 280 (100) | 245 (100) | 75 (100) | 39 (98) | 639 (99.8) |
| Median (Q1, Q3) number of observation visits conducted among those with no SUMs and >=1 observation visit completed | 49 (39, 61) | 19 (17, 21) | 20 (18, 22) | 4 (3, 8) | 22 (18, 47) |
| Median (Q1, Q3) observations of TSU among those with no SUMs and >=1 observation visit completed | 0 (0, 1) | 0 (0, 0) | 0 (0, 0) | 0 (0, 1) | 0 (0, 0) |

Table S11. Percent of intervention and control participants who ever moved during the trial, including use of biomass by intervention participants during moves and use of clean fuels by control participants during moves.

| | Guatemala | | India | | Peru | | Rwanda | | Total | |
|---|---|---|---|---|---|---|---|---|---|---|
| | Intvn | Cntrl | Intvn | Cntrl | Intvn | Cntrl | Intvn | Cntrl | Intvn | Cntrl |
| n | 400 | 400 | 398 | 399 | 394 | 402 | 392 | 404 | 1584 | 1605 |
| n (%) households that ever moved | 65 (16) | 77 (19) | 96 (24) | 98 (25) | 116 (29) | 131 (33) | 71 (18) | 60 (15) | 348 (22) | 366 (23) |
| n (%) Intervention participants who moved to house where biomass is used at all | 13 (3) | --- | 52 (13) | --- | 10 (3) | --- | 4 (1) | --- | 79 (5) | --- |
| Median (Q1, Q3) days spent by intervention participants in house where biomass is used at all | 104 (83, 121) | --- | 153 (86.5, 223.5) | --- | 86.5 (26, 168) | --- | 94 (64, 122) | --- | 136 (71, 215) | --- |
| n (%) Control participants who moved to house where clean fuel is used exclusively | --- | 2 (0.5) | --- | 7 (2) | --- | 42 (10) | --- | 0 | --- | 51 (3) |
| Median (Q1, Q3) days spent by control participants in house where clean fuel is used exclusively | --- | 506 (471, 540) | --- | 147 (18, 309) | --- | 136 (84, 220) | --- | 0 | --- | 141 (79, 246) |

The most frequent reasons for moves included visiting the mother's or mother-in-law's house, moving to a new personal residence, and moving to a seasonal residence. Visiting the mother's or mother-in-law's house was the most common reason for moves in Guatemala, India, and Peru, while moving to a new personal residence was most common in Rwanda. Reasons were similar between intervention and control participants. Across countries, control participants who moved to a house where clean fuel was used exclusively spent a median (Q1, Q3) of 141 (79, 246) days in the new or temporary home. Intervention participants who moved to a home where biomass is used at all spent a median (Q1, Q3) of 136 (71, 215) days in the new or temporary home, but only 29% (n=23) reported being the primary cook in that home.

Table S12. Demographic characteristics of participants by group, by country and overall.

| | Guatemala | | India | | Peru | | Rwanda | | Total | |
|---|---|---|---|---|---|---|---|---|---|---|
| | Intvn | Cntrl | Intvn | Cntrl | Intvn | Cntrl | Intvn | Cntrl | Intvn | Cntrl |
| n | 400 | 400 | 398 | 399 | 394 | 402 | 392 | 404 | 1584 | 1605 |
| Age: mean (SD) | 24.5 (4.4) | 25.0 (4.5) | 24.0 (3.7) | 23.9 (3.9) | 25.6 (4.3) | 25.4 (4.6) | 27.3 (4.3) | 27.3 (4.5) | 25.3 (4.4) | 25.4 (4.5) |
| Mother's highest level of education: n (%) | | | | | | | | | | |
| None or primary school incomplete | 189 (47) | 192 (48) | 128 (32) | 155 (39) | 15 (4) | 20 (5) | 145 (37) | 191 (47) | 477 (30) | 558 (35) |
| Primary school complete or secondary school incomplete | 160 (40) | 152 (38) | 116 (29) | 111 (28) | 131 (33) | 103 (26) | 151 (39) | 167 (41) | 558 (35) | 533 (33) |
| Secondary/ vocational school complete or some college | 51 (13) | 56 (14) | 154 (39) | 133 (33) | 247 (63) | 279 (69) | 96 (24) | 46 (11) | 548 (35) | 514 (32) |
| # Household Members: mean (SD) | 5.3 (2.7) | 5.1 (2.6) | 3.8 (1.6) | 3.8 (1.5) | 4.5 (1.7)[†] | 4.7 (1.8) | 3.5 (1.5) | 3.5 (1.5) | 4.3 (2.0)[‡] | 4.3 (2.0) |
| Owns household assets: n (%) | | | | | | | | | | |
| Color television | 169 (42) | 188 (47) | 290 (73) | 301 (75) | 246 (62) | 260 (65) | 66 (17) | 34 (8) | 771 (49) | 783 (49) |
| Radio | 153 (38) | 151 (38) | 57 (14) | 52 (13) | 287 (73) | 304 (76) | 234 (60) | 214 (53) | 731 (46) | 721 (45) |
| Mobile phone | 361 (90) | 370 (93) | 326 (82) | 327 (82) | 376 (95) | 388 (97) | 319 (81) | 310 (77) | 1382 (87) | 1395 (87) |
| Bicycle | 45 (11) | 53 (13) | 60 (15) | 61 (15) | 146 (37) | 162 (40) | 112 (29) | 133 (33) | 363 (23) | 409 (25) |
| Bank account | 99 (25) | 98 (25) | 355 (89) | 359 (90) | 93 (24) | 86 (21) | 147 (38) | 85 (21) | 694 (44) | 628 (39) |

[†] n=393; [‡] n=1583
